# Sinonasal Adenocarcinoma: Update on Classification, Immunophenotype and Molecular Features

**DOI:** 10.1007/s12105-016-0694-9

**Published:** 2016-02-01

**Authors:** Ilmo Leivo

**Affiliations:** Department of Pathology and Forensic Medicine, University of Turku, Turku, Finland

**Keywords:** Sinonasal adenocarcinoma, Salivary-type adenocarcinoma, Intestinal-type adenocarcinoma, Nonintestinal-type adenocarcinoma, Immunohistochemistry, Molecular pathology, Wood dust exposure

## Abstract

Adenocarcinomas of the sinonasal tract may originate from respiratory surface epithelium or the underlying seromucinous glands. These malignancies are divided into salivary-type adenocarcinomas and non-salivary-type adenocarcinomas. The latter are further divided into intestinal-type and nonintestinal-type adenocarcinomas. This review provides an update on tumor classification, differential diagnostic considerations and molecular features, as well as new adenocarcinoma entities in the sinonasal area.

## Salivary-Type Adenocarcinomas

Sinonasal salivary-type carcinomas arise from the seromucinous glands and surface epithelium of the nasal cavity and paranasal sinuses [1]. They comprise 5–10 % of sinonasal adenocarcinomas [2].

**Fig. 1:**
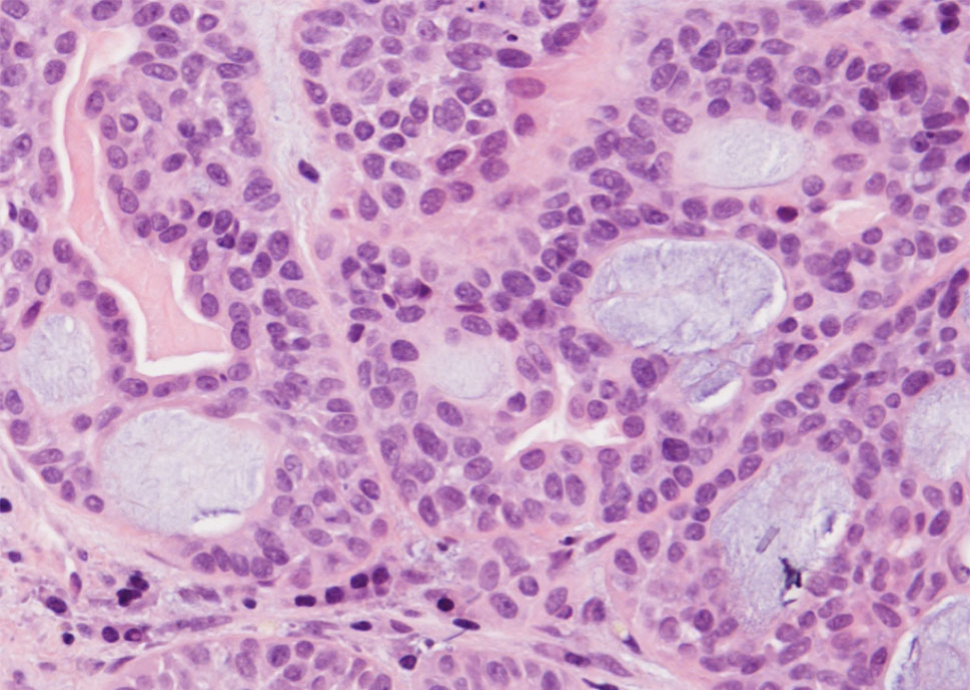
Adenoid cystic carcinoma of nasal cavity. Classic cribriform pattern with bland nuclear morphology. H–E stain ×400

The histopathological appearances of these tumors are for the most part similar to those of carcinomas and adenomas arising in major and minor salivary glands. Most of the tumor types that occur in major and minor glands also occur in the sinonasal area, with the exception of Warthin tumor and purely sebaceous salivary tumors [3]. Tumor types include pleomorphic adenoma, myoepithelioma, adenoid cystic carcinoma, mucoepidermoid carcinoma, acinic cell carcinoma, myoepithelial carcinoma, epithelial-myoepithelial carcinoma, salivary duct carcinoma, basal cell adenocarcinoma, polymorphous low-grade adenocarcinoma, carcinoma ex-pleomorphic adenoma, adenocarcinoma, NOS, and others. In the sinonasal tract, pleomorphic adenoma is the most frequent salivary-type tumor [1]. Adenoid cystic carcinoma (AdCC) is the most common salivary-type carcinoma, and the second most common sinonasal malignancy overall after squamous cell carcinoma, and it represents 10–18 % of all sinonasal malignancies [2, 4] (Fig. 1). AdCC usually occurs in the maxillary sinus or the nasal cavity [4]. Tumor type-specific gene rearrangements such as *MYB*-*NFIB* have been described in AdCC also in the sinonasal tract [5]. Long-term prognosis of AdCC is poor due to local spread. Mucoepidermoid carcinoma is less common representing around 5 % of sinonasal glandular tumors [2, 6]. Acinic cell carcinoma, epithelial-myoepithelial carcinoma, polymorphous low-grade adenocarcinoma, adenocarcinoma, NOS, and others are even more rare. Furthermore, rare sinonasal carcinomas have displayed both AdCC-like histological features with surface squamous dysplasia and an HPV-association (particularly of types 33 and 35), but not the *MYB* gene rearrangement frequent in AdCC of other organ sites [5].

Awareness of the possibility of salivary-type tumors in the sinonasal tract is important when diagnosing neoplastic lesions in this area. The differential diagnoses of salivary-type carcinomas include intestinal-type adenocarcinoma and nonintestinal-type adenocarcinoma. Furthermore, salivary-type malignancies with clear cell features such as hyalinizing clear cell carcinoma must be differentiated from renal cell carcinoma metastatic to the sinonasal tract. On the other hand, differential diagnosis of a metastatic renal cell carcinoma should also include the rare sinonasal renal cell-like adenocarcinoma of the sinonasal tract [7].

The treatment of salivary-type tumors is complete surgical removal. The 5-year survival rates have ranged from 40 to 60 %, with the poorest results in AdCC [3, 4]. Postoperative radiotherapy has been recommended.

### (Non-salivary-Type) Surface Epithelial Adenocarcinomas

The WHO classifies sinonasal adenocarcinomas of surface epithelial origin in intestinal and nonintestinal types [8].

### Intestinal-Type Adenocarcinoma

Intestinal-type adenocarcinoma (ITAC) is the second most common type of sinonasal adenocarcinoma after AdCC. It is composed of growth patterns that resemble carcinomas or adenomas of intestinal origin, or it may mimic normal histology of the intestinal mucosa [9, 10]. ITACs occur mostly in males in a wide age range with a mean around 50 to 64 years. The tumors are most often localized in the ethmoid sinus (40 %), followed by the nasal cavity (25 %) and the maxillary antrum (20 %). ITACs are aggressive malignancies, and may spread to adjacent structures including the orbit, the pterygopalatine fossa, the infratemporal fossa and the cranial cavity.

A remarkable association has been identified between long-term exposure to wood dusts and the occurrence of ITAC [11–15]. In woodworking industries, workers with occupational exposure to hardwood dusts may show incidences 1000 times those of the general population. Occupational wood dust exposure has been observed in ca. 20 % of cases. Interestingly, the highest incidences are seen in woodworkers in the furniture industry where hardwoods, particularly beech and oak, are used [13, 16]. Also, ITAC is frequent in long-term wood dust exposure in woodworkers who lay hardwood floors. Also, occupational exposure to dusts in the shoe and leather industry [17] and in textile manufacture, as well as to chromium and nickel, have been incriminated [18]. The carcinogenic compounds have not been identified, but a possible etiologic role for tannins has been suspected [18]. The cumulative exposure time to wood dusts in patients with ITAC has been 40–43 years [9]. ITACs associated with dust exposure occur predominantly in the ethmoid sinus, while sporadic ITACs often arise in the maxillary antrum.

ITACs mimic the appearance of the mucosa in normal and neoplastic large and small intestine. Based on histologic parameters, Barnes subdivided ITACs into five categories: papillary, colonic, solid, mucinous, and mixed types [9]. The classification of Kleinsasser and Schroeder [19] subdivides ITACs into papillary-tubular cylindrical cell type (corresponding to papillary, colonic, and solid types), alveolar goblet cell type, signet-ring cell type (corresponding to mucinous type), and transitional cell type (corresponding to mixed type). The histologic subtypes have been found to correlate with clinical behavior [9, 18, 19].

**Fig. 2:**
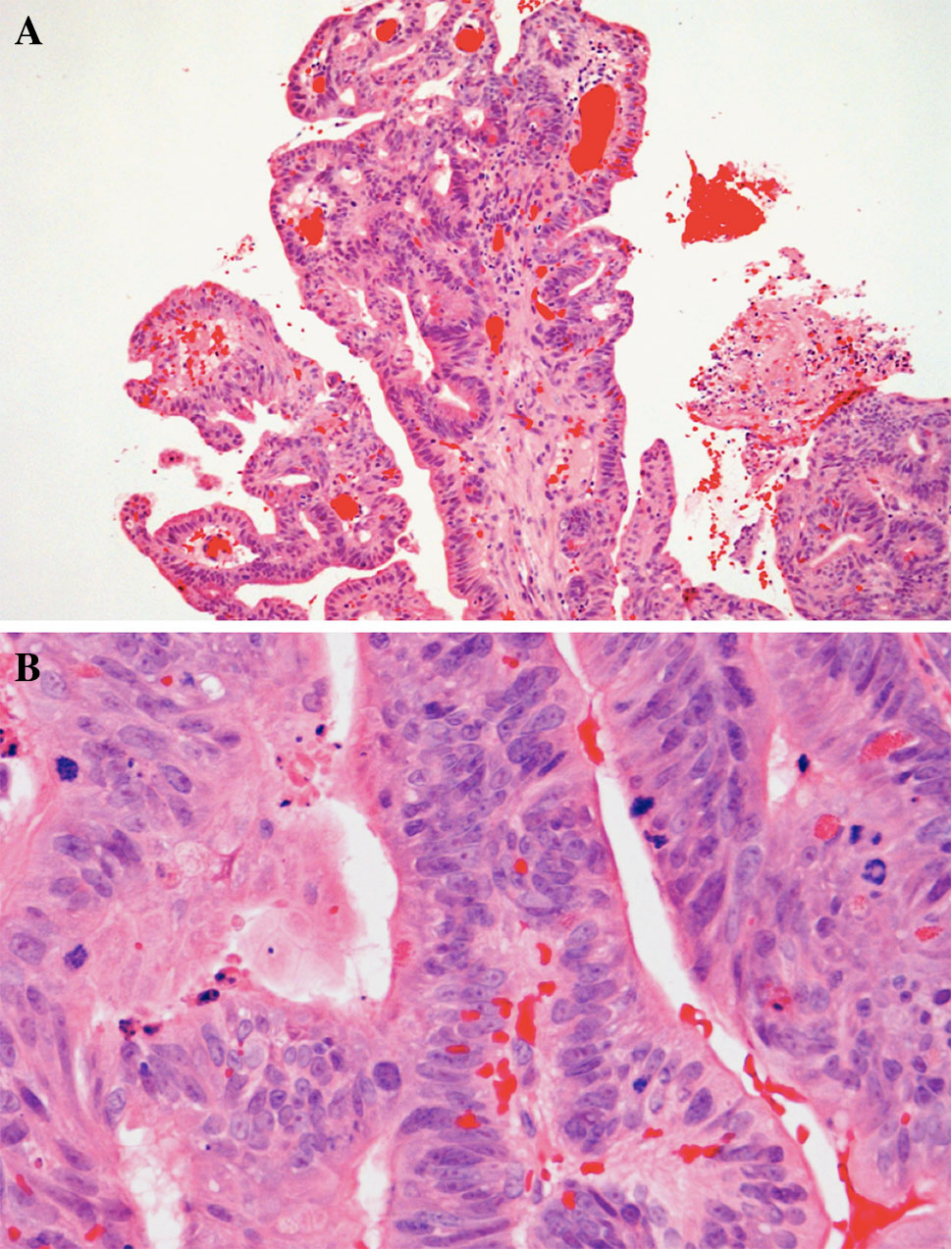
**a** Intestinal-type adenocarcinoma, papillary growth pattern. The pattern is composed of papillary projections and glandular and tubular structures. H–E stain ×250. **b** Tumor of Fig. 3a. The nuclei are elongated, irregular and often hyperchromatic. There is some nuclear piling. Cellular form is mostly cylindrical. There are many mitotic figures. H–E stain ×400

The papillary type (ca. 18 % of ITACs) shows a prominent papillary architecture with few tubular areas (Fig. 2a). Tumors contain columnar goblet cells mimicking intestinal adenomas (Fig. 2b). Occasionally, papillary ITACs may recapitulate normal intestinal mucosa with normal-looking villi, and with the specialized cell types (goblet, resorptive, Paneth, and argentaffin cells) and the muscularis mucosae [20].

**Fig. 3:**
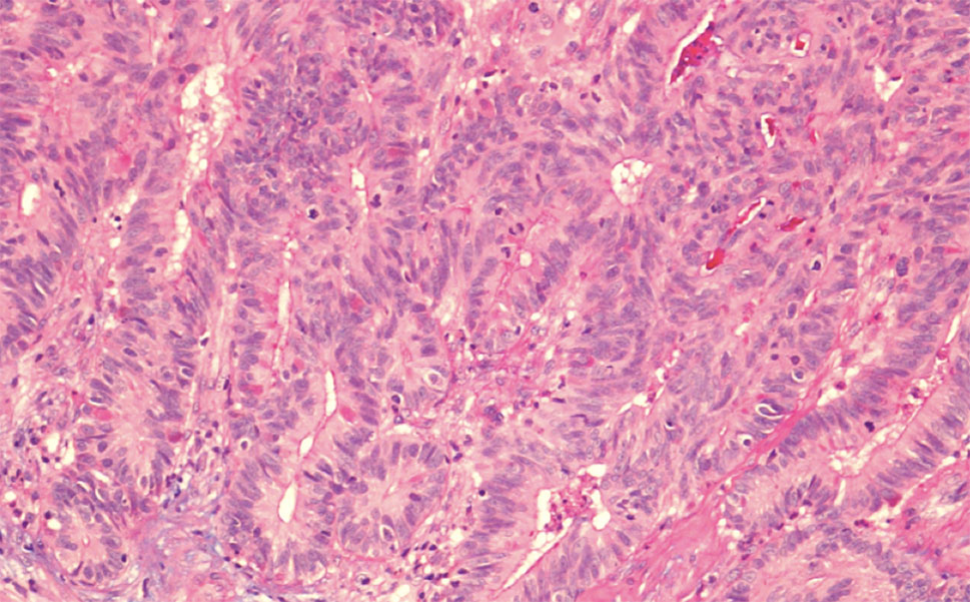
Intestinal-type adenocarcinoma, colonic growth pattern. Glandular structures and some trabecular areas. High mitotic activity. H–E stain ×250

**Fig. 4:**
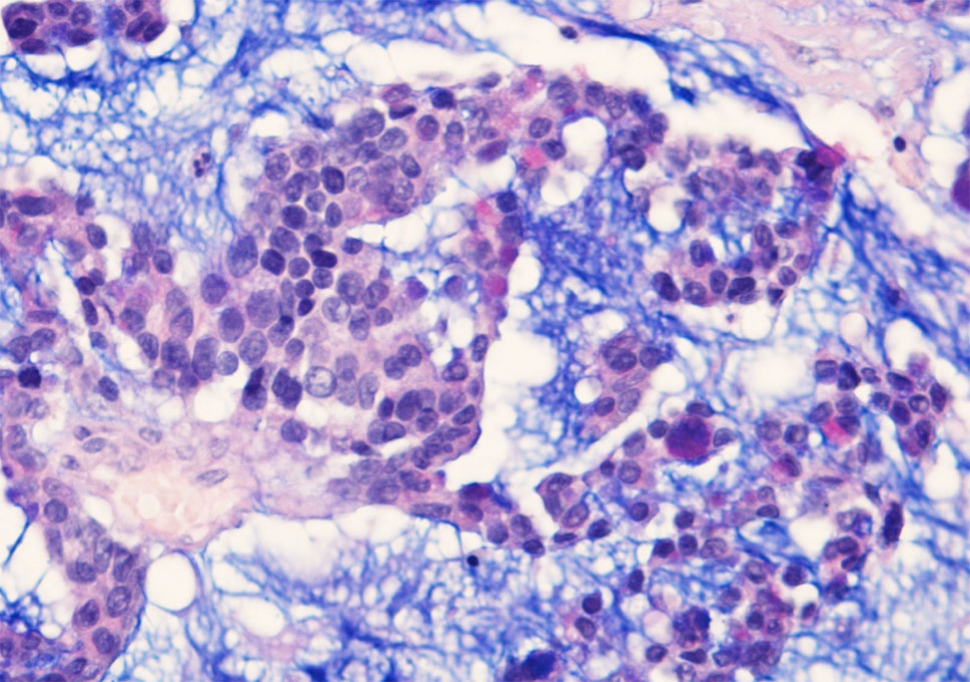
Intestinal-type adenocarcinoma, mucinous growth pattern. Clusters of tumor cells contain a few goblet-type cells, and are suspended in a pool of Alcian Blue-positive mucin. Alcian-Blue PAS stain ×400

The colonic type is the most frequent ITAC (40 %). It displays glandular, tubular and trabecular architecture and few papillae, and resembles a conventional colorectal adenocarcinoma (Fig. 3). Crowded columnar cells with variation in nuclear size and shape line the tumor glands. Intra- and extracellular mucin and few goblet cells may be seen. These tumors are often widely invasive. The solid type comprises less differentiated ITACs with predominantly solid growth patterns. The mucinous type displays mucin-filled glands, or cell clusters that float in pools of extracellular mucin (Fig. 4) and often contain signet-ring cells. Mucinous ITACs closely mimic the mucinous variants of colorectal adenocarcinoma. The mixed type contains a mixture of the preceding types.

**Fig. 5:**
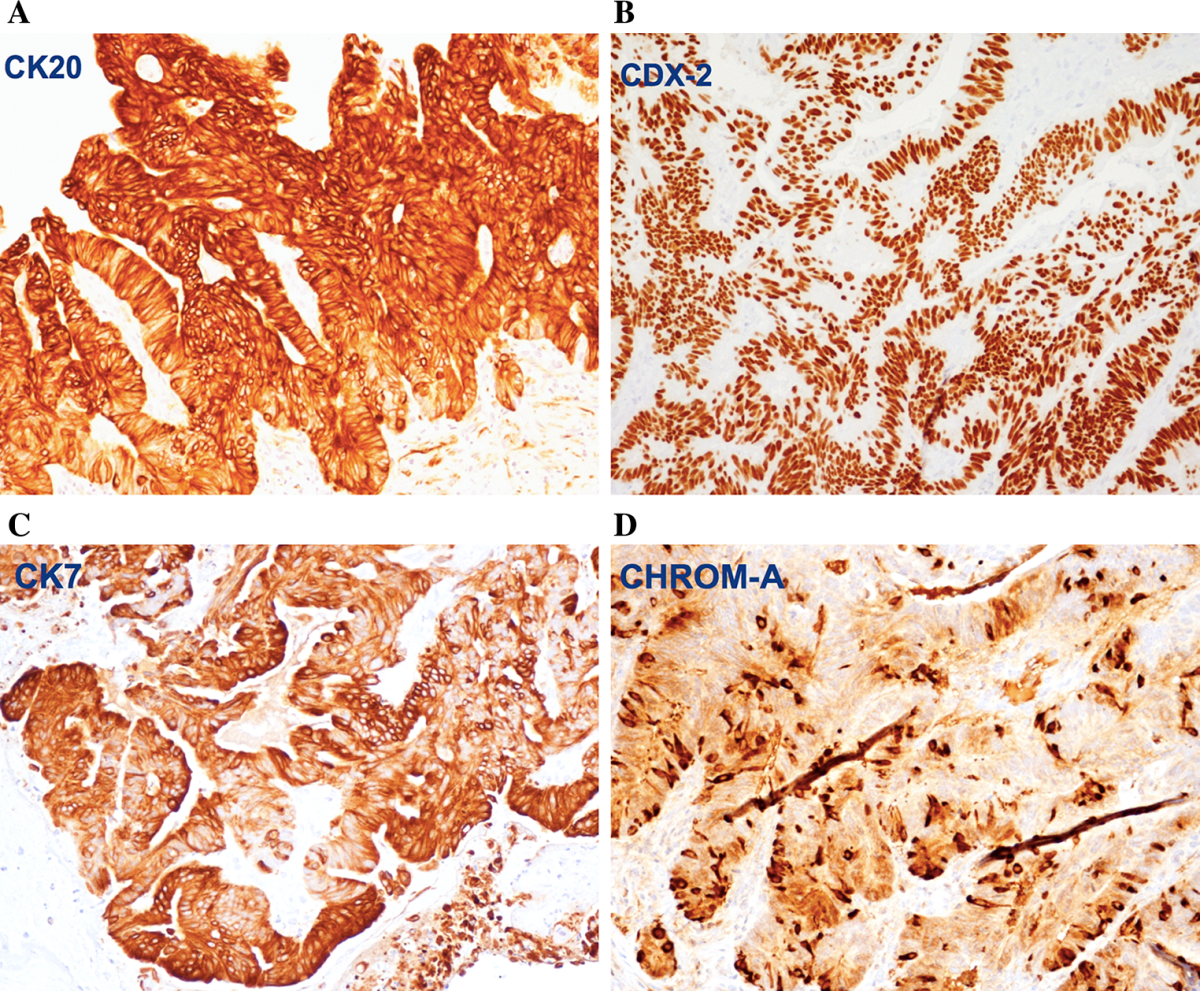
Immunohistochemical staining of intestinal-type adenocarcinoma, papillary growth pattern, for **a** CK20, **b** CDX-2, **c** CK7, and **d** chromogranin A. Peroxidase conjugated ABC Kit (Dako) ×250

The immunophenotype of ITAC includes staining for CK20 (Fig. 5a), CDX2 (Fig. 5b), villin, and MUC2, and variable positivity for CK7 (Fig. 5c) [21–23]. Focal chromogranin A (Fig. 5d) and synaptophysin may be seen in the neuroendocrine cells. A subset of ITACs, mostly in woodworkers, expressed high levels of EGFR protein [24]. In contrast to colorectal carcinomas, activating mutations of K-RAS and BRAF in the signal route of EGFR are rare [25–27]. This suggests possibilities for anti-EGFR therapies in ITAC. Other molecular studies indicate preserved expression of mismatch repair proteins, β-catenin and E-cadherin [28], and overexpression of MET protein [29]. Annexin A1 and A2 were down-regulated in ITAC [30]. High prevalence of *TP53* mutations was seen in sinonasal carcinoma with work-related exposure to wood dust [31].

Differential diagnosis of ITAC includes metastatic gastrointestinal carcinoma and sinonasal low-grade nonintestinal adenocarcinoma. On grounds of histology and immunophenotype, colonic or mucinous ITAC cannot be distinguished with certainty from colorectal carcinoma metastatic to the sinonasal tract. Both ITACs and colorectal carcinomas express CK20, CDX-2, MUC2, and villin, while the presence of CK7 may be suggestive of ITAC. However, colonoscopy or colorectal radiographic studies should be employed to rule out primary colorectal carcinoma in case of an intestinal-type tumor in the sinonasal tract. Furthermore, while CDX-2 is helpful for diagnosing ITAC, it is not absolutely specific, as it can be expressed also in sinonasal undifferentiated carcinomas and rarely in salivary-type adenocarcinomas. More specific for ITAC than the expression of CDX-2 is the expression of CK20 [32].

The treatment of ITAC is surgical resection varying from lateral rhinotomy and partial maxillectomy to total maxillectomy. Surgery may be combined with radiotherapy.

The behavior of ITAC is that of a high-grade malignancy. In a study of 213 ITACs, 50 % of patients developed recurrences, 8 % had cervical lymph node metastases, 13 % had distant metastases, and 60 % died of their disease [9]. Well differentiated papillary ITACs have an indolent course, but patients with solid and mucinous ITACs have an untoward outcome [9, 18, 19].

### Nonintestinal-Type Adenocarcinoma

These adenocarcinomas display histopathologic features of neither sinonasal intestinal-type adenocarcinomas nor salivary-type adenocarcinomas, and they are divided in high-grade and low-grade types [8].

**Fig. 6:**
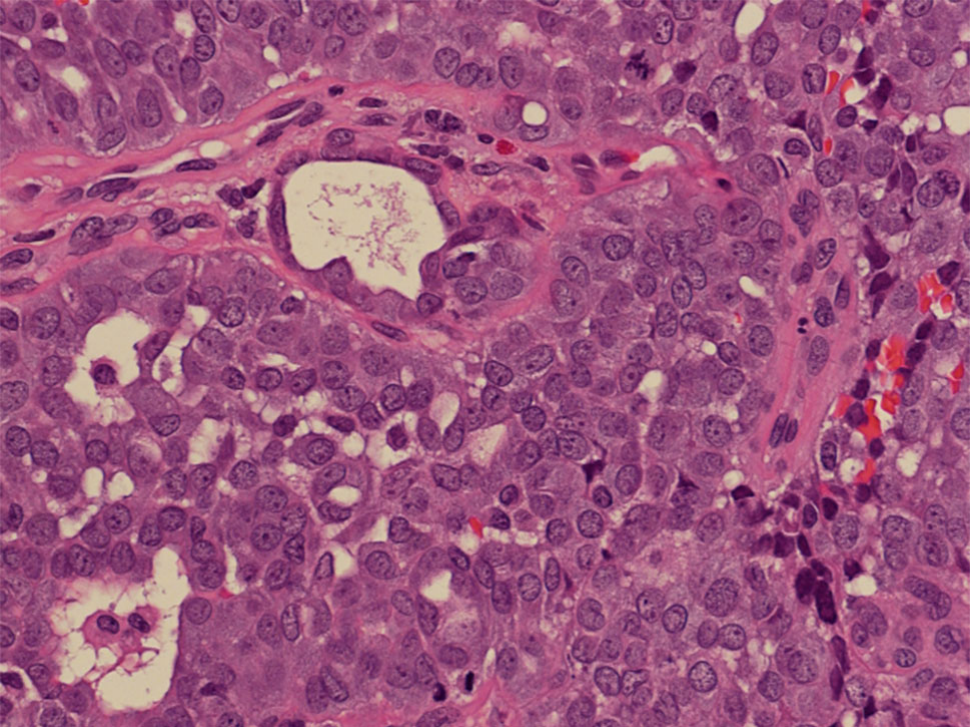
High-grade nonintestinal-type adenocarcinoma of nasal cavity. This high-grade tumor is poorly differentiated and displays atypical mitotic figures. It has areas of glandular differentiation, but does not exhibit intestinal or salivary-type features. H–E stain ×400

*High*-*grade nonintestinal*-*type adenocarcinomas* are rare malignancies of the sinonasal tract. Patients are frequently males with a wide age range and a mean around 60 years. Histopathologically, these tumors have been reported to display a diversity of morphologic patterns such as blastomatous, apocrine, oncocytic/mucinous, poorly differentiated/undifferentiated, and others [33]. Their nuclei tend to be pleomorphic and there is mitotic activity (Fig. 6). These tumors were reported to lack CDX-2 and CK20 immunoreactivity [33]. The heterogeneous features of these tumors may overlap with those of other malignancies of this area, and consequently other primary and metastatic malignancies must be carefully ruled out before making this diagnosis. In particular, differential diagnosis from salivary-type adenocarcinoma, NOS may be very challenging. The prognosis of high-grade nonintestinal-type adenocarcinomas is poor [33, 34].

*Low*-*grade nonintestinal*-*type adenocarcinomas* are uncommon (13 % of sinonasal adenocarcinomas) and occur mostly in the ethmoid sinus, the nasal cavity, and the maxillary sinuses. The age range is wide with a mean of 37–53 years. Synonyms in the literature include terminal tubulus adenocarcinoma [35], sinonasal tubulopapillary low-grade adenocarcinoma [36, 37], sinonasal low-grade adenocarcinoma [34], and sinonasal seromucinous adenocarcinoma [38]. These carcinomas have no known association with environmental carcinogens.

**Fig. 7:**
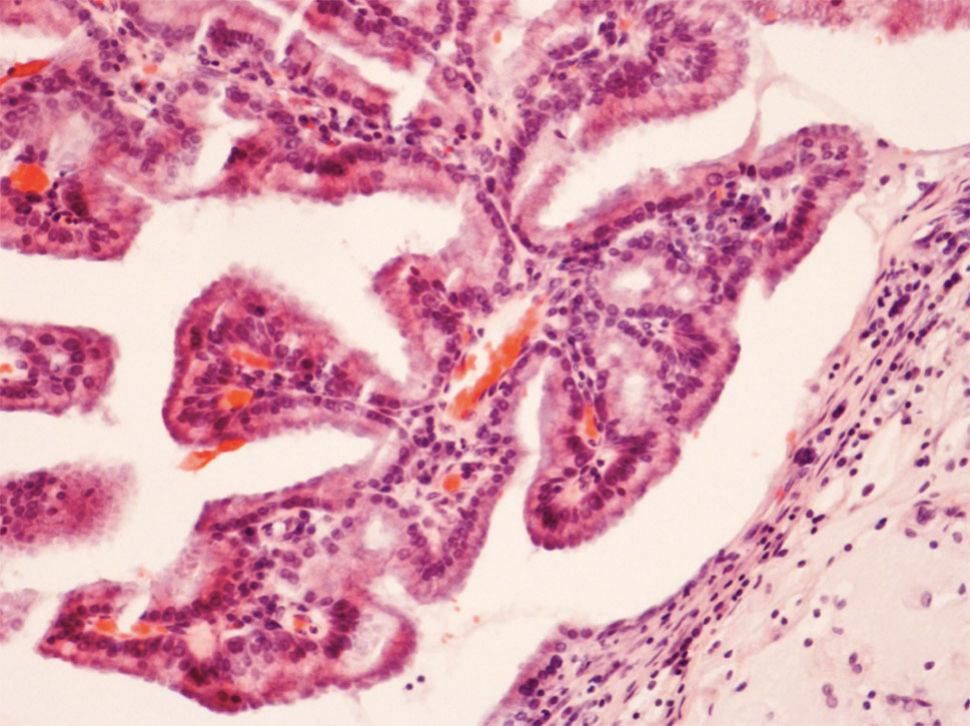
Low-grade nonintestinal-type adenocarcinoma of nasal cavity. A complex papillary growth pattern with some glandular structures. A single layer of bland columnar cells line the papillae. Nuclear pleomorphism is minimal and no mitotic figures are seen ×400

**Fig. 8:**
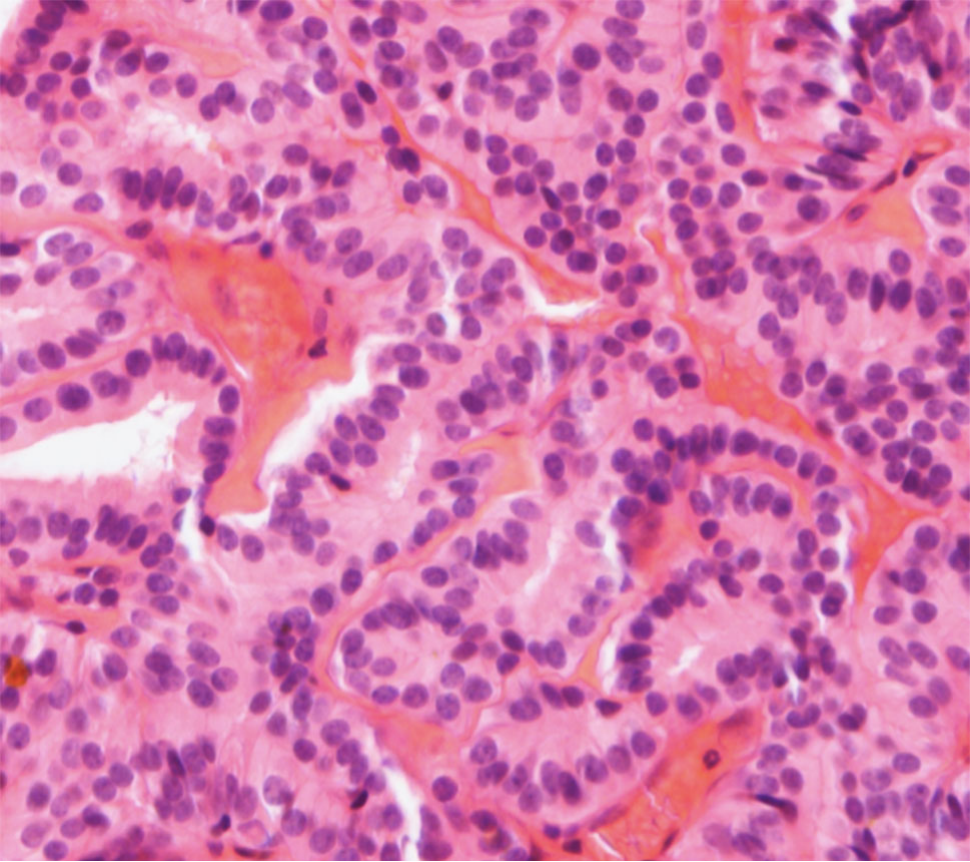
Low-grade nonintestinal-type adenocarcinoma, tubulopapillary pattern. A papillary and tubular growth pattern with a single layer of cuboidal to columnar cells with round uniform nuclei, indistinct nucleoli and eosinophilic cytoplasm. H–E stain ×400

Histopathologically, low-grade nonintestinal-type carcinomas exhibit varied architectural forms with exophytic papillae and tubular or glandular patterns [36, 39, 40] (Fig. 7). Trabecular, cribriform, clear cell and mucinous patterns have also been reported. The papillae and glands are usually lined by a single layer of uniform columnar or cuboidal cells with only minor cytologic aberrations. They show a bland low-grade cytology with round, uniform nuclei and inconspicuous nucleoli (Fig. 8). Mitotic figures are very rare. In these bland tumors, complexity of the growth pattern and local invasive growth are findings supporting a diagnosis of malignancy. Immunohistochemically, they are constantly positive for CK7, but usually negative for CK20 and CDX-2. Some 20 % of these tumors associate with sinonasal seromucinous hamartomas or respiratory epithelial adenomatoid hamartomas [39, 41, 42]. Due to positivity for markers of seromucinous differentiation such as DOG1, SOX10, and S-100, a subset of these carcinomas was called sinonasal seromucinous adenocarcinomas [42].

Differential diagnosis of low-grade nonintestinal-type adenocarcinoma includes ITAC, acinic cell carcinoma, oncocytic Schneiderian papilloma and, rarely, metastatic papillary carcinoma of the thyroid. Differentiation of these low-grade carcinomas from ITAC is highly important in view of the distinct behaviors of the two neoplasms. The defined growth patterns and high nuclear grade of ITAC usually allow for a clear-cut differential diagnosis. Immunohistochemically, low-grade nonintestinal-type adenocarcinomas remain negative for CK20, CDX-2 and MUC2. However, rare ITACs that resemble normal intestinal mucosa may present a source of confusion. Even such bland-looking ITACs are potentially high-grade malignancies. Oncocytic Schneiderian papillomas may be confused with low-grade nonintestinal-type adenocarcinomas although the epithelium of papillomas is multilayered and does not contain true glandular lumina [34]. Metastatic papillary carcinoma of the thyroid can be distinguished with stains for TTF-1 and thyroglobulin.

The treatment of low-grade nonintestinal-type adenocarcinoma is complete surgical removal, and radical procedures are seldom needed. Radiotherapy is optional. The disease is usually localized, but local recurrences are possible [34]. Metastasis is unusual, and death of disease is rare. The overall prognosis of the patients is favorable.
